# Two simple assays for assessing the seeding activity of proteopathic tau

**DOI:** 10.3389/fnagi.2023.1073774

**Published:** 2023-04-06

**Authors:** Fei Liu, Ruozhen Wu, Nana Jin, Dandan Chu, Jianlan Gu, Yunn Chyn Tung, Zhihao Hu, Cheng-Xin Gong, Khalid Iqbal

**Affiliations:** ^1^Department of Neurochemistry, Inge Grundke-Iqbal Research Floor, New York State Institute for Basic Research in Developmental Disabilities, New York, NY, United States; ^2^Key Laboratory of Neuroregeneration of Jiangsu and Ministry of Education of China, Nantong University, Nantong, Jiangsu, China

**Keywords:** Alzheimer’s disease, tau, tau propagation, seeding activity, tau pathology

## Abstract

The regional distribution of neurofibrillary tangles of hyperphosphorylated tau aggregates is associated with the progression of Alzheimer’s disease (AD). Misfolded proteopathic tau recruits naïve tau and templates its misfolding and aggregation in a prion-like fashion, which is believed to be the molecular basis of propagation of tau pathology. A practical way to assess tau seeding activity is to measure its ability to recruit/bind other tau molecules and to induce tau aggregation. Based on the properties of proteopathic tau, here we report the development of two simple assays to assess tau seeding activity ----- capture assay *in vitro* and seeded-tau aggregation assay in cultured cells. In the capture assay, proteopathic tau was applied onto a nitrocellulose membrane and the membrane was incubated with cell lysate containing HA-tagged tau_151-391_ (HA-tau_151-391_). The captured tau on the membrane was determined by immuno-blots developed with anti-HA. For the seeded-tau aggregation assay, HEK-293FT cells transiently expressing HA-tau_151-391_ were treated with proteopathic tau in the presence of Lipofectamine 2000 and then lysed with RIPA buffer. RIPA-insoluble fraction containing aggregated tau was obtained by ultracentrifugation and analyzed by immuno-blot developed with anti-HA. To validate these two assays, we assessed the seeding activity of tau in the middle frontal gyrus, middle temporal gyrus and basal forebrain of AD and control brains and found that AD, but not control, brain extracts effectively captured and seeded tau_151-391_ aggregation. Basal forebrain contained less phospho-tau and tau seeding activity. The levels of captured tau or seeded-tau aggregates were positively correlated to the levels of phospho-tau, Braak stages and tangle sores. These two assays are specific and sensitive and can be carried out in a regular biomedical laboratory setting by using routine biochemical techniques.

## Introduction

Neurofibrillary tangles (NFTs) consisting of hyperphosphorylated tau aggregates are a hallmark of Alzheimer’s disease (AD) and related neurodegenerative tauopathies. It was found that tau lesion of NFTs in AD brain starts from the trans-entorhinal–entorhinal areas, from there to hippocampus, limbic areas, and finally in the associative and then primary neocortical areas (Braak and Braak, 1991, 1995), which associates strongly with the progression of cognitive impairment (Braak and Braak, 1995). Recently, similar stages of tau pathology were shown by tau tracer retention measured with positron emission tomography (PET) (Johnson et al., 2016; Scholl et al., 2016; Schwarz et al., 2016). Thus, regional distribution of tau pathology associates with the progression of AD.

Misfolded proteopathic tau is known to recruit and template naïve tau misfolding and aggregation in prion-like fashion. *In vitro*, cytosolic and hyperphosphorylated soluble tau isolated from AD brain recruits recombinant tau to form paired helical filaments in a non-saturable manner (Alonso et al., 1994, 1996). Tau aggregates/filaments either isolated from AD brain or generated *in vitro* induce tau aggregation (Friedhoff et al., 1998; von Bergen et al., 2000). In cultured cells, tau aggregates enter the cells and template the aggregation of intracellular soluble tau (Frost et al., 2009; Guo and Lee, 2011; Kfoury et al., 2012). *In vivo*, injection of tau aggregates into mouse brain induces tau aggregation in the injection site and associated distant brain regions (Clavaguera et al., 2009, 2013; Iba et al., 2013; Hu et al., 2016). The seeding activity of tau from AD brain is correlated with Braak stages positively and with MMSE (Mini-Mental State Examination) scores negatively and precedes overt tau pathology (Furman et al., 2017). Seeded tau aggregation underlies the spreading of tau pathology. Thus, assessment of tau seeding activity in human specimens is relevant to the clinical outcome. In addition, the tau seeding activity assays also offer a platform for drug screening and for measuring efficacy of treatment that target tau pathology.

Tau seeding activity has been assessed by *in vitro* seed amplification assays, such as the Real-Time Quaking-Induced Conversion-based (RT-QuIC-based) assay (Dujardin et al., 2020; Metrick et al., 2020), by cell-based assays in cultured cells, e.g., the fluorescence resonance energy transfer (FRET)-biosensor assay (Holmes et al., 2014), and by *in vivo* seed amplification assays (Clavaguera et al., 2009; Lathuiliere and Hyman, 2021). We previously found that truncated tau_151-391_ is highly prone to being captured and templated to aggregation by oligomeric tau derived from AD brain (AD O-tau) (Gu et al., 2020). By using tau_151-391_, we introduce the development of two simple assays with routine biochemical techniques, an *in vitro* tau capture assay and in cultured cells a seeded-tau aggregation assay, for the assessment of tau seeding activity and validated the assays using the extracts from middle frontal gyrus (MFG), middle temporal gyrus (MTG), and basal forebrain (BFB) of AD and control brains. We found that the brain extracts from all AD cases except for one whose brain tau was not hyperphosphorylated, but not from control brains, captured tau *in vitro* and seeded tau aggregation in cultured cells. BFB displayed less phospho-tau and less seeding activity compared with MTG and MFG. It was found that tau seeding activity was positively correlated with the levels of hyperphosphorylated tau, Braak stages and tangle scores. Of special note, these two assays can be performed in a regular biomedical laboratory and do not require any special equipment or technique. In addition to measuring tau seeding activity, these assays can also offer a platform for studying the roles of tau mutations and post-translational modifications in prion-like aggregation, for drug screening and for measuring the efficacy of treatments that target tau pathology.

## Materials and methods

### Human brain tissue

Frozen autopsied brain tissues from five clinically diagnosed and histopathologically confirmed AD cases and five control cases (Table 1) were obtained from the Sun City Health Research Institute Brain Donation Program (Sun City, AZ, United States). The control cases were cognitively normal, without any significant neuropathological findings that could contribute to cognitive symptoms. Tissues of three brain regions (MTG, MFG, and BFB) were used in the present study. The use of autopsied frozen human brain tissue was in accordance with the National Institutes of Health guidelines and was exempted by the Institutional Review Board (IRB) of the New York State Institute for Basic Research in Developmental Disabilities because “the research does not involve intervention or interaction with the individuals” nor “is the information individually identifiable.” The brain tissue samples were stored at −80°C until used.

**Table 1:** Human brain tissues of Alzheimer’s disease and control cases used in this study*.

Diagnosis	Case #	Age (year)	Gender	PMI (hr)	Braak Stage^a^	Tangle scores^b^
AD	00–18	89	F	2.33	V	8.66
AD	00–33	73	F	2	V	15
AD	00–22	60	M	3.33	VI	15
AD	00–13	87	M	2.4	V	14.5
AD	00–29	60	F	3.5	VI	15
		73.8 ± 14.02		2.71 ± 0.66		13.63 ± 2.79***
Con	00–34	85	M	3.16	II	4.25
Con	03–28	80	M	2.16	I	1
Con	03–50	91	M	3.33	III	3.5
Con	03–63	83	F	3.25	II	0.75
Con	00–49	86	F	2.5	III	5
		85 ± 4.06		2.88 ± 0.52		2.90 ± 1.93

*Frontal cerebral cortices from AD and control cases were obtained from the Sun City Health Research Institute Brain Donation Program (Sun City, AZ, United States). PMI, post-mortem interval. ^a^Neurofibrillary pathology was staged according to Braak and Braak (1995). ^b^Tangle score was a density estimate and was designated none, sparse, moderate, or frequent (0, 1, 2, or 3 for statistics), as defined according to CERAD Alzheimer’s disease criteria (Mirra et al., 1991). Five areas (frontal, temporal, parietal, hippocampal, and entorhinal) were examined, and the scores were totaled for a maximum of 15. ***p < 0.0001, AD *vs* Con.

Brain tissue was homogenized in cold homogenate buffer (20 mM Tris–HCl), pH 8.0, 0.32 M sucrose, 1 mM Na_3_VO_4_, 50 mM NaF, 10 mM glycerophosphate, 10 mM β-mercaptoethanol (β-ME), 5 mM MgSO_4_, 1 mM 4-(2-aminoethyl) benzenesulfonyl fluoride hydrochloride (AEBSF), 1 mM EDTA, and 10 μg/mL each of leupeptin, aprotinin, and pepstatin. The homogenates were centrifugated at 10,000 × g at 4^ο^C for 10 min and the supernatants were saved for analyses of tau phosphorylation and seeding activity.

### Plasmids, antibodies, and other reagents

pCI/HA-tau_151-391_ and pCI/HA-tau_1-441_ were constructed as previously described (Gu et al., 2020). The primary antibodies used in the present study are listed in Table 2. Horseradish peroxidase (HRP)-conjugated secondary antibodies were purchased from Jackson ImmunoResearch Laboratories (West Grove, PA, United States). Alexa 488-conjugated-anti-mouse IgG was obtained from Thermo Fisher Scientific Corporation (Waltham, MA, United States).

**Table 2:** Primary antibodies used in the present study.

Antibody	Type	Species	Specificity	Source/reference (cat/lot)
43D	Mono-	M	Tau (6–18)	In-house/BioLegend (816601) (Li et al., 2019)
71C11	Mono-	M	Tau (2 N)	In-house/BioLegend (816801)
R134d	Poly-	R	Tau	In-house (Li et al., 2019)
92e	Poly-	R	Tau	In-house (Li et al., 2019)
Tau-5	Mono-	M	Tau (210–230)	Millipore (MAB361/1816394)
AT8	Mono-	M	pS202/pThr205-tau	Thermo Scientific (MN1020/PI205175)
PHF-1	Mono-	M	pSer396/Ser404-tau	Dr. Peter Davies (Greenberg et al., 1992)
Anti-HA	Mono-	M	HA	Sigma (H9658/112 M4841)
Anti-HA	Poly-	R	HA	Sigma (H6908/115M4872V)
Anti-GAPDH	Poly-	R	GAPDH	Sigma (G9545/015M4824V)

Mono-, monoclonal; Poly-, polyclonal; p-, phosphorylated; M, Mouse; R, Rabbit.

### Cell culture and transfection

Human cervix epithelial cell line (HeLa) and human embryonic kidney cell line (HEK-293FT) were maintained with Dulbecco’s modified Eagle’s medium (DMEM) (Thermo Fisher Scientific) supplemented with 10% fetal bovine serum (FBS) (Thermo Fisher Scientific), 100 μg/mL streptomycin, and 100 U/mL penicillin (Thermo Fisher Scientific) in a humidified atmosphere containing 5% CO_2_ at 37°C. All transfections were carried out with FuGENE HD (Promega, Madison, WI, United States) according to the instructions provided by manufacturer. Empty vectors were used as mock controls for the corresponding transfection.

### Preparation of oligomeric tau from AD brain

As we described previously, AD O-tau was isolated from the cerebral cortex of frozen autopsied AD brains (Hu et al., 2016; Li et al., 2021). Briefly, 10% brain homogenate prepared in the buffer as described above was centrifuged at 27,000 × g for 30 min at 4°C. The supernatant was further centrifuged at 235,000 × g for 45 min at 4°C. The pellet, AD O-tau–enriched fraction, was collected and washed three times. AD O-tau was resuspended in saline, probe-sonicated for 10 min at 20% power, and stored at −80°C until used. The levels of tau and phospho-tau were analyzed by a mixture of pan-tau antibodies, R134d and 92e (Li et al., 2021).

### Generation of tau_151-391_ aggregates

Tau_151-391_ was overexpressed in HEK-293FT cells. The cells were lysed in RIPA (radio immunoprecipitation assay) buffer (50 mM Tris–HCl, pH 8.0, 150 mM NaCl, 0.5% sodium deoxycholate, 0.1% SDS, and 1% Nonidet P-40 (NP-40) and containing 1 mM Na_3_VO_4_,50 mM NaF, 1 mM AEBSF, and 10 μg/mL each of leupeptin, aprotinin, and pepstatin). RIPA-insoluble fraction was yielded by 75,000 × g for 30 min and washed with PBS following with the centrifugation. The RIPA-insoluble fraction containing aggregated tau was used to treat new HEK-293FT/tau_151-391_ cells for 42 h. RIPA-insoluble tau_151-391_ aggregates were obtained by repeating the treatment up to three times.

### Western blots

Cultured cells were lysed directly in 1x Laemmli sample buffer containing 1 mM AEBSF and 10 μg/mL each of leupeptin, aprotinin, and pepstatin and then heated as above. Brain extracts were adjusted to 1x Laemmli sample buffer. Samples were heated in a boiling-water bath for 5 min and subjected to protein concentration measurement using the Pierce™ 660 nm Protein Assay Kit (Thermo Fisher Scientific). Proteins in the samples were separated by SDS-PAGE and transferred onto a polyvinylidene fluoride membrane (MilliporeSigma, Burlington, MA, United States). After blocking with 5% fat-free milk in Tris-buffered saline (TBS), the membrane was incubated with primary antibody (Table 2) in 5% fat-free milk in TBS containing 0.1% NaN_3_ overnight at room temperature (RT), washed with TBST (TBS with 0.05% Tween 20), and incubated with HRP-conjugated 2nd antibody in 5% fat-free milk in TBS, washed with TBST, incubated with the enhanced chemiluminescence (ECL) substrate (Thermo Fisher Scientific), and exposed to HyBlot CL^®^ autoradiography film (Denville Scientific Inc., Holliston, MA, USA) or detected by an iBright Imager (Thermo Fisher Scientific). Specific immunosignal was quantified by using the Multi Gauge software V3.0 from Fuji Film (Minato, Tokyo, Japan).

### Immuno-dot blots

Various amounts of samples were applied onto a nitrocellulose (NC) membrane (Schleicher and Schuell, Keene, NH, United States) at 5 μL per grid of 7 × 7 mm size in duplicate or triplicate. After drying in a 37°C oven for 1 h, the membrane was developed with specific antibodies as described above for Western blots.

### Tau capture assay *in vitro*

Cell lysate containing hemagglutinin-tau (HA-tau): HA-tau_151-391_ (in the numbering of the 441–amino acid isoform of human tau) was overexpressed in HEK-293FT cells for 48 h. The cells were probe-sonicated in cold lysate buffer containing 50 mM Tris–HCl, pH7.4, 0.15 M NaCl, 50 mM NaF, 1 mM Na_3_VO_4_, 1 mM EDTA, 1 mM AEBSF and 10 μg/mL (each of leupeptin, aprotinin, and pepstatin) for 2 min with 20% amplitude at 4°C. Cell lysate was centrifuged for 5 min at 10,000 × g. The supernatant was stored at −80°C until used.

Capturing tau from the cell lysate: Various amounts of samples containing proteopathic tau were applied onto an NC membrane in triplicate as described above for immuno-dot blots. The membrane was dried, blocked with 5% fat-free milk in TBS and incubated with cell lysate containing HA-tau overnight at RT. Captured tau on the membrane was developed with anti-HA as described above for immuno-blots. Several exposures were taken. The non-saturated images with high ratio of signal and noise were quantified by densitometry as described above.

### Seeded-tau aggregation assay in cultured cells

Cultured HEK-293FT cells in 24-well plate in triplicate or quadruplicate were transiently transfected with pCI/HA-tau_151–391_, or pCI/HA-tau_1-441_. Six hours after transfection, proteopathic tau sample in 25 μL of Opti-MEM containing 2–3% Lipofectamine 2000 was added to the culture. The cells were lysed in RIPA buffer containing 1 mM Na_3_VO_4_, 50 mM NaF, 1 mM AEBSF, and 10 μg/mL each of leupeptin, aprotinin, and pepstatin for 20 min on ice or in 1% Sarkosyl in buffer (50 mM Tris–HCl, pH7.4, 150 mM NaCl, 1 mM EDTA, 1 mM Na_3_VO_4_, 50 mM NaF, 1 mM AEBSF, and 10 μg/mL each of leupeptin, aprotinin, and pepstatin) by agitation at RT for 1 h after 42 h treatment. The cell lysate was centrifuged at 75,000 × g at 4°C for 30 min. The supernatant and the pellet were saved as RIPA- or sarkosyl–soluble and -insoluble fractions, respectively. The pellet was washed with PBS. Levels of tau in the insoluble and soluble fractions were analyzed by immuno-blots developed with anti-HA.

### Depletion of tau from AD brain extract

Tau antibodies 71C11, AT8, Tau5, and PHF-1, and as a control, mouse IgG (mIgG) were pre-coupled by incubating with protein G-agarose for 6 h at RT. After washing with TBS, the antibody-coupled beads were incubated with the same volume of AD brain extract at 4°C overnight. The supernatant was collected as tau-depleted AD extract.

### Denaturing AD O-tau by boiling in Laemmli buffer

Various amounts of AD O-tau were applied onto an NC membrane as described above. After drying, the membrane was boiled in TBS or Laemmli buffer for 5 min and followed by above tau capture assay.

### Statistical analysis

In this study, GraphPad Prism 8 (GraphPad Software Inc., San Diego, CA, United States) was used for statistical analyses. Data are presented as mean ± standard deviation (SD). Shapiro–Wilk test and/or Kolmogorov–Smirnov test for normality were used to assess data distribution. Unpaired two-tailed Student’s *t* test (data with normal distribution) or Mann–Whitney test (data with non-normal distribution) was used for the comparison between two groups. One-way or two-way analysis of variance (ANOVA) was followed by Tukey’s or by Sidak’s multiple comparisons. For correlation analysis, Pearson (data with normal distribution) or Spearman (data with non-normal distribution) correlation and linear or non-linear regression were performed.

## Results

### Evaluation of tau seeding activity by the tau capture assay

Misfolded proteopathic protein recruits the naïve protein, templates the misfolding and aggregation, which underlies the propagation of the proteopathies. The recruitment of naïve protein is the initial step for the templated aggregation. Based on the property of proteopathic protein templated aggregation, we designed a tau capture assay to assess the recruitment ability of proteopathic tau by using AD O-tau as the proteopathic seeds, which we previously charactered as the most potent seeds in seeding tau aggregation in AD brain (Li et al., 2021).

**Figure 1:**
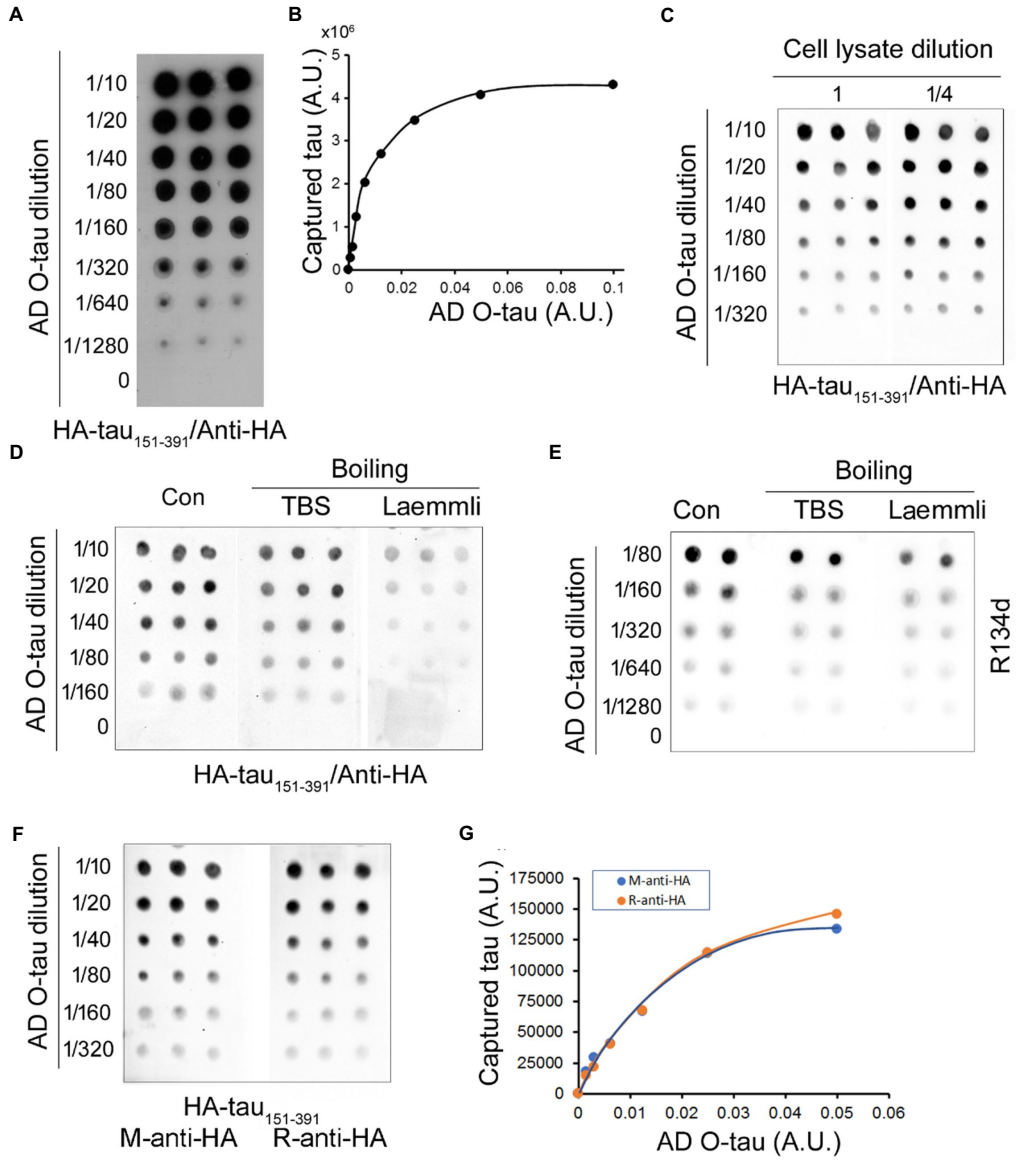
Assessment of tau seeding activity by the tau capture assay. **(A,B)** AD O-tau captured tau_151-391_ effectively. Various amounts of AD O-tau were applied onto NC membrane in triplicate. The membrane was blocked with 5% milk and incubated with the lysate of HEK-293FT cells expressing HA-tau_151-391_. Captured tau was developed with anti-HA followed by HRP-2_nd_ antibody and ECL **(A)**. The levels of captured tau were plotted against amounts of AD O-tau **(B)**. **(C)** Limited dilution of HEK-293FT cell lysates did not affect the levels of captured tau. NC membranes applied with various amounts of AD O-tau in triplicate were incubated with the lysate or ¼ diluted cell lysate containing HA-tau_151-391_ after blocking. Captured tau was analyzed as described in panel **A.** **(D,E)** Denaturing by boiling in Laemmli buffer partially inhibited the capability of AD O-tau to capture tau_151-391_. NC membranes applied with AD O-tau in duplicate or triplicate were boiled in Laemmli buffer or TBS for 5 min and then subjected to the capture assay **(D)** or immuno-dot blots **(E)**. **(F,G)** Anti-HA from mouse and from rabbit detected captured tau similarly. NC membrane applied with AD O-tau in triplicate was incubated with the cell lysate containing HA-tau_151-391_. Captured tau was developed with monoclonal mouse anti-HA or polyclonal rabbit anti-HA **(F)** and plotted against the amounts of AD O-tau **(G)**. A.U., arbitrary unit.

Five μL of serially diluted AD O-tau from 1:10 to 1:1280 were applied onto an NC membrane. The membrane was incubated with the cell lysate containing HA-tau_151-391_ after blocking with 5% milk. The recruited tau was detected with monoclonal anti-HA followed by HRP-conjugated secondary antibody as for immuno-dot blot described previously (Gu et al., 2020). We found that tau_151-391_ from the cell lysate was captured by AD O-tau in a dose-dependent manner (Figures 1A,B). Theoretically, this assay requires over-saturation of HA-tau_151-391_ in the cell lysate used. We thus also determined the HA-tau_151-391_ level captured from the cell lysate after 1:4 dilution. We found similar amounts of tau_151-391_ being captured from the 1:4 diluted lysate as from the undiluted lysates (Figure 1C). These results suggested the over-saturation of HA-tau_151-391_ in the cell lysate, indicating that small variations of HA-tau_151-391_ expression efficiency will not affect the accuracy of this assay.

β-sheet is required for templated aggregation by proteopathic protein (Woerman et al., 2016). Laemmli buffer containing SDS and β-ME is widely used for denaturing proteins for Western blots. NC membrane applied with various amounts of AD O-tau was boiled in the Laemmli buffer or TBS for 5 min, and then subjected to the tau capture assay as described above. We found that the amount of captured tau was obviously reduced by boiling the membrane in the Laemmli buffer, but not in TBS (Figures 1D,E), suggesting that denaturing by boiling in Laemmli buffer reduces the capability of AD O-tau to capture tau_151-391_.

Tau_151-391_ was tagged with HA at its N-terminus. Thus, the captured tau was evaluated by mouse monoclonal or rabbit polyclonal anti-HAs. We found similar immunoactivity with mouse anti-HA and rabbit anti-HA (Figures 1F,G), suggesting that both antibodies displayed similar sensitivity and thus can be used for this assay.

### Assessment of tau seeding activity in AD brain by the capture assay

**Figure 2:**
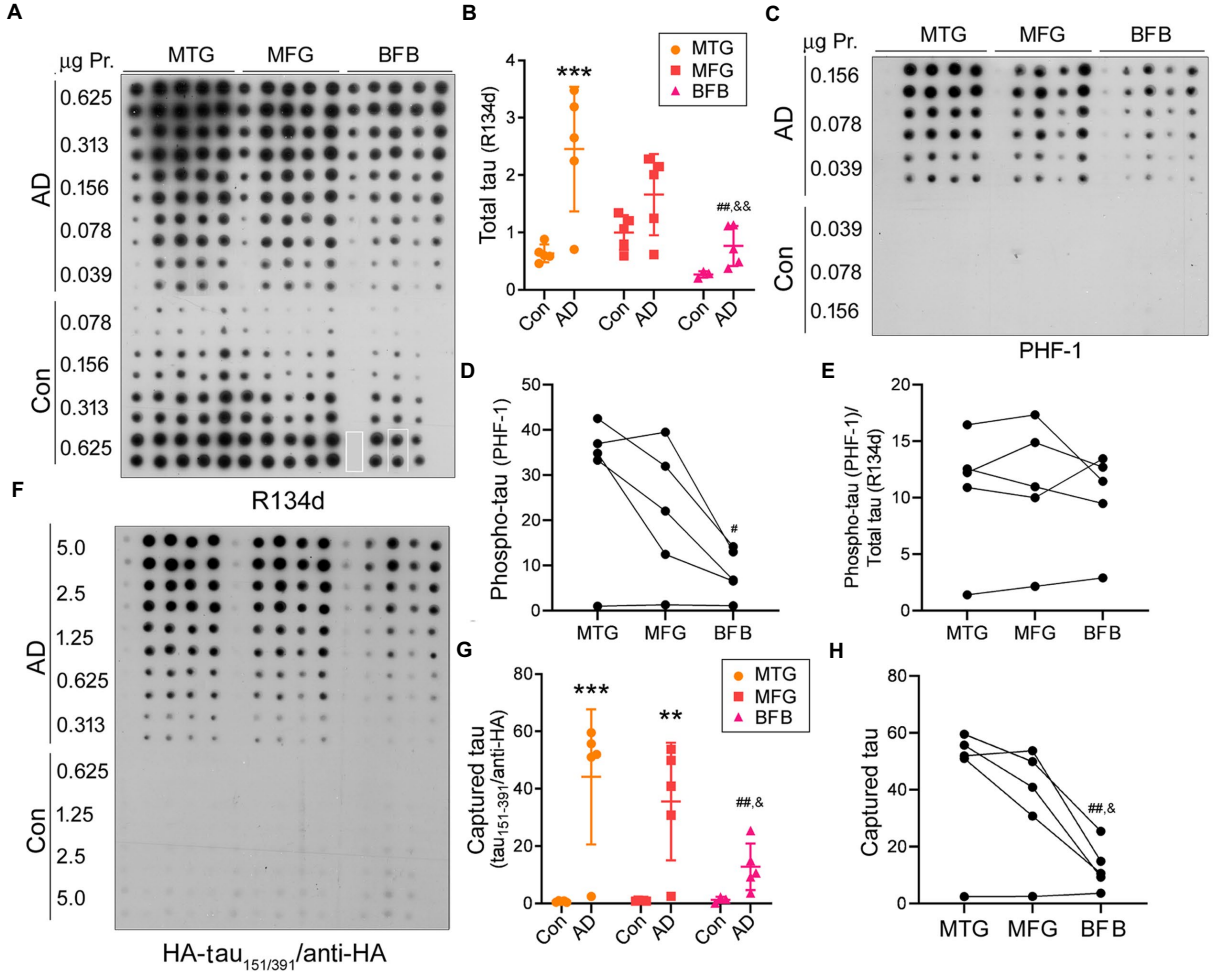
Tau seeding activity in AD brain assessed by the capture assay. **(A,B)** Increased tau level in MTG, MFG, and BFB of AD compared with the corresponding regions of control cases. Tau in brain extracts from three regions of AD and control cases was analyzed by immuno-dot blot developed with pan-tau antibody R134d **(A)**. BFB samples of control case 1 and case 5 were unavailable and therefore are not included here. The highlighted rectangles in R134d blot indicate the exchange of grids. Levels of tau are presented as scatter plots and mean ± SD **(B)**. **(C–E)** Hyperphosphorylated tau at PHF-1 site in AD brains. Phospho-tau in the brain extracts was analyzed by immuno-dot blots developed with PHF-1 **(C)**. The levels of phospho-tau **(D)** and phosphorylation of tau (phospho-tau/total tau) **(E)** are presented crossing three brain regions of AD cases. **(F–H)** AD, but not control, brain extracts were able to capture tau_151-391_. Various amounts of brain extracts from the three regions of AD and control cases were applied onto an NC membrane in duplicate and subjected to the tau capture assay **(F)**. Levels of captured tau are presented as scatter plots and mean ± SD **(G)** and crossing three brain regions **(H)**. *, compared with corresponding region of control; ^#^, compared with MTG; ^&^, compared with MFG; ^#^,^&^, *p* < 0.05; **,^##^,^&&^, *p* < 0.01; ***, *p* < 0.001.

Using the capture assay, we assessed tau seeding activity in three brain regions, the middle frontal gyrus (MFG), middle temporal gyrus (MTG), and basal forebrain (BFB), of AD and control cases. Brain homogenates were centrifuged at 10,000 x g for 10 min at 4°C to yield the extracts. Serially diluted brain extracts were applied onto the NC membranes. The membrane was blotted with pan-tau antibody R134d (Figure 2A) or phospho-tau antibody PHF-1 (Figure 2C) to determine their levels. We found increased levels of total tau (Figure 2B) and phospho-tau (Figure 2D) in the three brain regions of AD cases, suggesting the accumulation of hyperphosphorylated tau in the three brain regions of AD. Compared with MTG, BFB extract expressed less total or phospho-tau (Figures 2B,D). However, tau phosphorylation resulting from normalization of phospho-tau with total tau was not significantly different among the three regions of AD brain (Figure 2E).

Parallelly, a membrane applied with various amounts (0.313–5 μg protein) of brain extracts was subjected to the tau capture assay by incubating the membrane with the cell lysate containing HA-tau_151-391_ as described above. We found that HA-tau_151-391_ was captured by the extracts of the three brain regions of AD, but not control cases (Figures 2F,G) and less HA-tau_151-391_ was captured by AD BFB extracts as compared to those of the two other brain regions (Figures 2G,H). These data suggest that tau in AD, but not in control brain displays the seeding activity and that tau seeding activity in AD brain can be evaluated by the capture assay.

### Association of tau seeding activity evaluated by the tau capture assay with tau pathology

**Figure 3:**
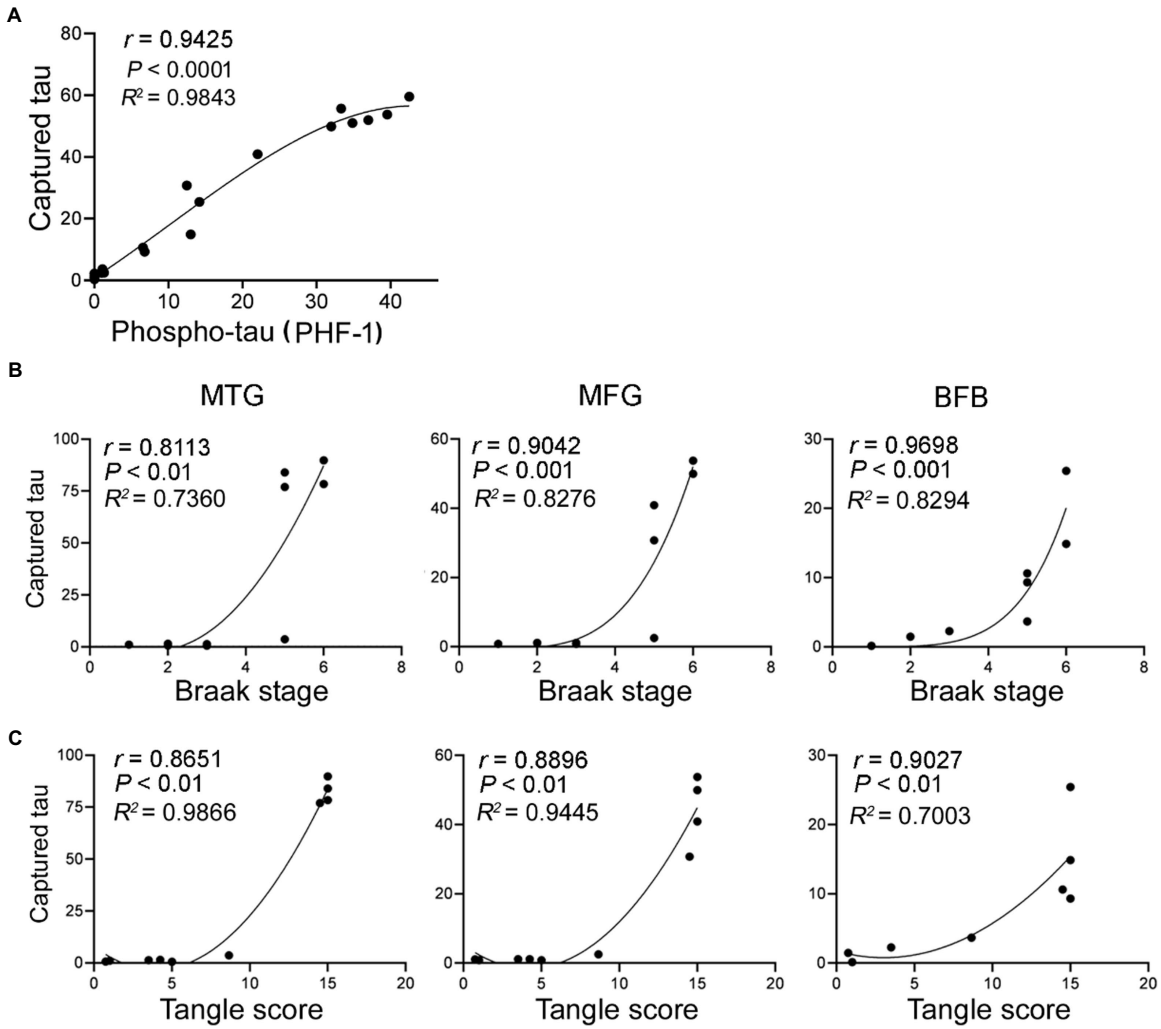
Tau seeding activity assessed by the capture assay is positively correlated with phospho-tau, Braak stage and tangle score. The levels of tau captured by brain extracts from the three brain regions of AD and control cases determined in Figure 2F were plotted against the levels of phospho-tau **(A)** as determined in Figure 2C, Braak stage **(B)** and tangle score **(C)**. The Spearman correlation and non-linear regression were analyzed.

In AD brain, tau is abnormally hyperphosphorylated at multiple sites (Zhou et al., 2018). To learn the relationship of tau seeding activity with tau phosphorylation, we performed the Spearman correlation analysis and non-linear regression. We found that levels of captured tau_151-391_ were highly positively correlated with the level of phospho-tau (Figure 3A), suggesting that tau seeding activity is associated with hyperphosphorylation of tau.

Hyperphosphorylated tau aggregates to form NFTs, a hallmark of AD and related tauopathies. Non-parametric correlation analyses suggested that the seeding activity in the three brain regions was positively correlated with the Braak stages (Figure 3B) and tangle sores (Figure 3C). Thus, tau seeding activity measured by using our assay in AD brain is associated with tau pathology.

### Seeded tau aggregation assay in cultured cells

Proteopathic tau enters the cells and templates tau aggregation, which underlies the propagation of tau pathology. We previously found that tau_151-391_ was prone to be templated to insoluble aggregates by AD O-tau (Gu et al., 2020). Based on the previous study, here we developed the seeded tau aggregation assay to analyze the ability of prion-like tau seeds to induce tau aggregation in cultured cells.

**Figure 4:**
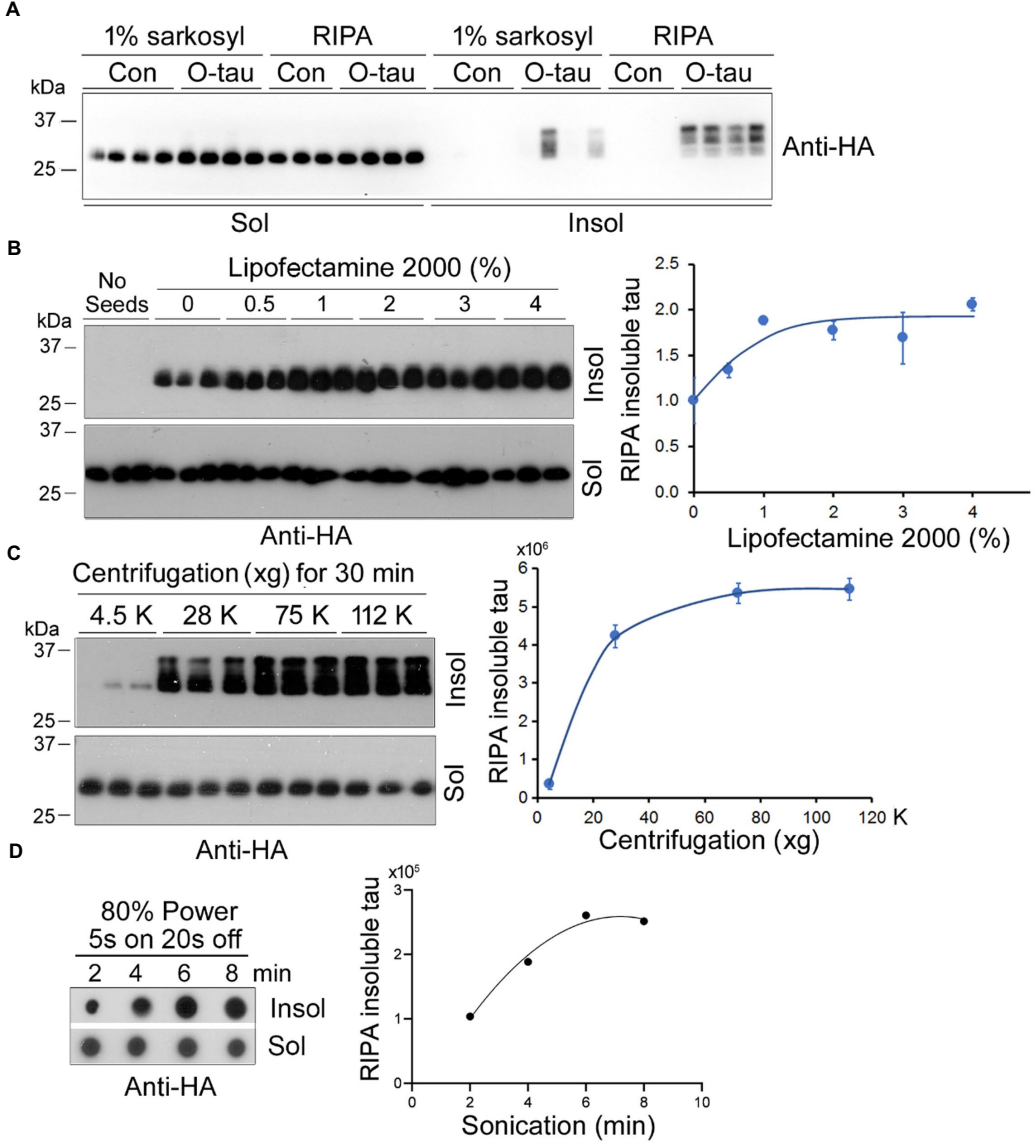
Optimization of the seeded tau aggregation assay. **(A)** RIPA buffer lysed the cells effectively. HEK-293FT cells in triplicate or quadruplicate were transiently expressed HA-tau_151-391_ and treated with AD O-tau (0.5 A.U.) 6 h after transfection. The cells were lysed in 150 μL RIPA buffer on ice for 20 min or in 1% sarkosyl at RT for 1 h by agitation. The soluble and insoluble fractions were obtained by ultracentrifugation and analyzed by Western blots with anti-HA. **(B)** Lipofectamine 2000 facilitated AD O-tau to seed tau_151-391_ aggregation. HEK-293FT cells expressing HA-tau_151-391_ in triplicate were cultured in 24-well-plate. AD O-tau (0.5 A.U.) was incubated with various concentrations of Lipofectamine 2000 in 25 μL of Opti-MEM for 5 min and then added into the cell culture. The cells were lysed with RIPA buffer. RIPA-soluble and RIPA-insoluble taus were analyzed by Western blots. RIPA-insoluble tau was plotted against the concentration of Lipofectamine 2000 used for delivering AD O-tau. **(C)** RIPA-insoluble tau was sedimented at 75,000 xg for 30 min. Cell lysates with RIPA buffer were centrifugated at various g for 30 min. RIPA-soluble and -insoluble taus were analyzed. RIPA-insoluble tau was plotted against centrifugal force. **(D)** Eight min bath sonication dissolved RIPA-insoluble tau. RIPA-insoluble fraction was sonicated for various times at 80% power, 5 s on and 3 s off and analyzed by immuno-dot blots developed with anti-HA. RIPA-insoluble tau was plotted against the sonication time.

Amyloid-type protein aggregates are detergent-insoluble. We cultured HEK-293FT cells in 24-well plate and transiently transfected the cells with pCI/HA-tau_151-391_. Six hours later, the cells were treated with 0.5 A.U. of AD O-tau for 42 h and lysed with 150 μL of RIPA buffer containing phosphatase and proteinase inhibitors on ice for 20 min or 1% Sarkosyl in buffer supplemented with phosphatase and proteinase inhibitors by agitation at RT for 1 h. After ultracentrifugation, tau in soluble and insoluble fractions was analyzed by Western blots developed with anti-HA. We found semi colloid of sarkosyl-lysates and similar levels of tau in RIPA and sarkosyl-soluble fractions (Figure 4A). In the cells without AD O-tau treatment, neither RIPA nor sarkosyl-insoluble tau was detectable (Figure 4A), suggesting seeded tau_151-391_ aggregation by AD O-tau. Of note, we found similar pattern and levels of RIPA-insoluble tau but various levels of sarkosyl-insoluble tau (Figure 4A). Thus, we chose RIPA buffer to lyse the cells.

Lipofectamine 2000 is known to facilitate the entry of proteopathic proteins into cells (Nonaka et al., 2010). To optimize the concentration of Lipofectamine 2000, HEK-293FT cells expressing HA-tau_151-391_ were treated with 0.5 A.U. of AD O-tau in 25 μL Opti-MEM containing various concentrations of Lipofectamine 2000 for 42 h and were lysed with RIPA buffer. RIPA-insoluble and -insoluble fractions were obtained by ultracentrifugation and analyzed by Western blots developed with anti-HA (Figure 4B). We found that Lipofectamine 2000 enhanced the function of AD O-tau in a dose-dependent manner and in cultured HEK-293FT cells 2–3% Lipofectamine 2000 was enough to AD O-tau to template tau aggregation (Figure 4B).

To optimize the centrifugal force for the sedimentation of aggregated tau, we centrifugated RIPA-cell lysate at various centrifugal forces for 30 min. The RIPA-soluble supernatant and the RIPA-insoluble pellet were analyzed by Western blots with anti-HA (Figure 4C). We found that 75,000 × g for 30 min was sufficient to sediment aggregated tau effectively (Figure 4C).

To dissociate the aggregated tau in the RIPA-insoluble fraction, we added PBS, sonicated for various times at 80% power with Fisherbrand™ Cup Horn 705 Sonic Dismembrator, and analyzed the tau by immuno-dot blots (Figure 4D). We found that 8 min sonication was able to dissolve the aggregated tau (Figure 4D).

Taking together, for the optimum templated tau aggregation assay, HEK-293FT cells in 24-well-plate transient-expressing HA-tau_151-391_ are treated with proteopathic tau seeds in 25 μL of 2–3% Lipofectamine 2000 in Opti-MEM for 42 h. The cells are lysed in RIPA buffer, RIPA-soluble and -insoluble fractions are obtained by 75,000 × g for 30 min. RIPA-insoluble fraction is washed with PBS following with 75,000 × g for 30 min and sonicated in Laemmli buffer or PBS for 8 min at 80% power. RIPA-insoluble aggregated tau is then analyzed by immuno-dot blots to determine the tau seeding activity.

**Figure 5:**
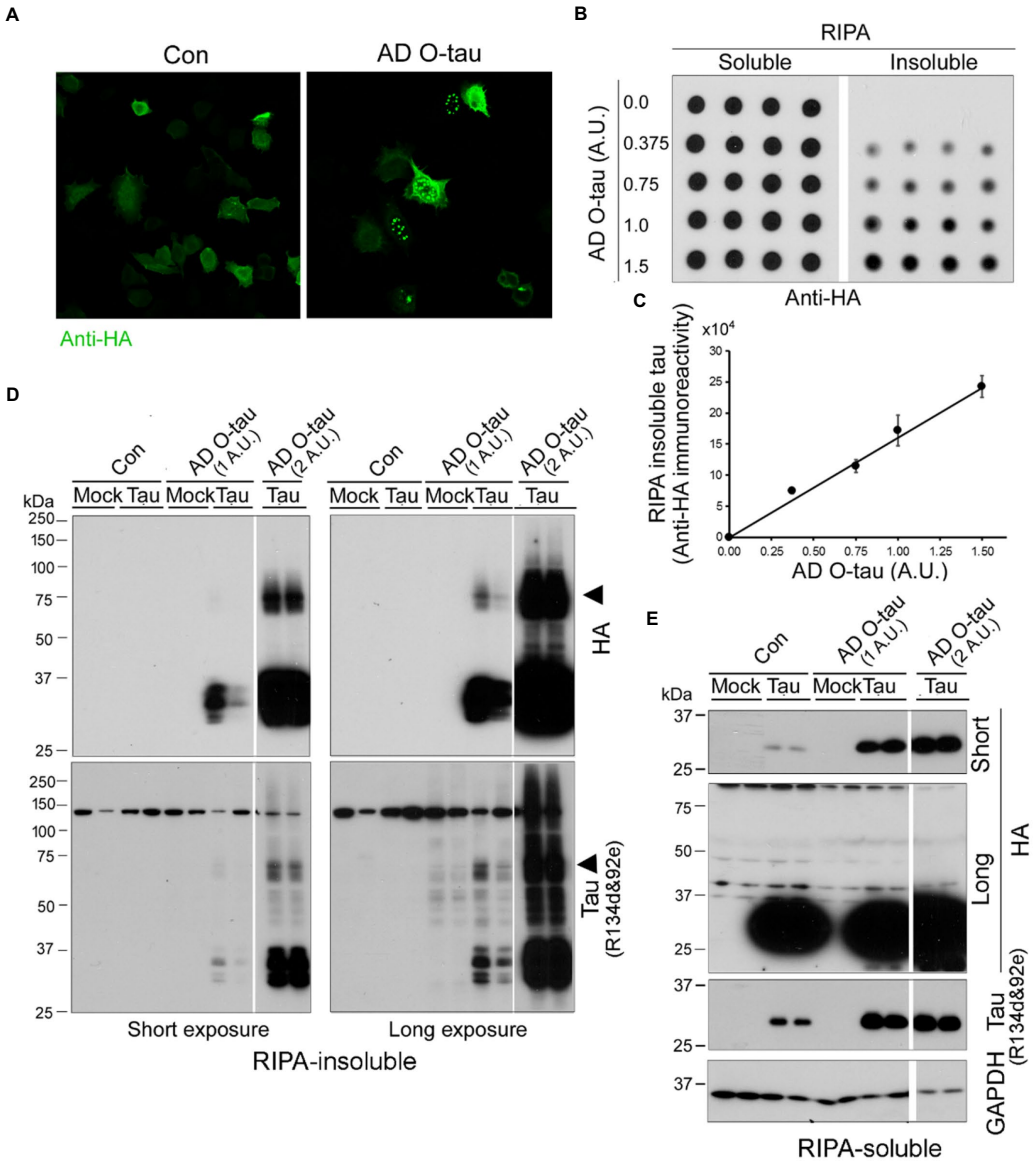
AD O-tau induces tau aggregation dose-dependently. **(A)** AD O-tau seeded tau_151-391_ aggregation in cultured HeLa cells. HeLa cells expressing HA-tau_151-391_ were treated with AD O-tau and immunostained with anti-HA 42 h after the treatment. **(B,C)** AD O-tau seeded tau aggregation dose-dependently. HEK-293FT cells expressing HA-tau_151-391_ in quadruplicate were treated with various amounts of AD O-tau. RIPA-insoluble and -insoluble taus were analyzed by immuno-dot blots developed with anti-HA **(B)** 42 h after treatment. RIPA-insoluble tau was plotted against amounts of AD O-tau **(C)**. **(D,E)** AD O-tau seeded tau aggregates displayed SDS- and β-ME-resistant high molecular weight tau (HMW-tau) species. HEK-293FT cells expressing HA-tau_151-391_ in duplicate were treated with AD O-tau. RIPA-insoluble **(D)** and -insoluble taus **(E)** were analyzed by Western blots developed with anti-HA or anti-pan-tau (a mixture of R134d and 92e), non-transfected cells are labeled as mock. Arrowhead indicates the SDS- and β-ME-resistant HMW-tau species.

To visualize tau aggregation templated by AD O-tau, we employed HeLa cells instead of HEK-293FT cells because the former are much large than the latter and are commonly employed to visualize protein aggregates. HeLa cells expressing HA-tau_151-391_ were treated with AD O-tau (1 A.U.) for 42 h. Tau aggregates were visualized by immunofluorescence staining with anti-HA. We found bright puncta in AD O-tau treated cells, but not in control cells (Figure 5A), suggesting that AD O-tau seeded tau_151-391_ aggregation. Interestingly, the seeded tau aggregates presented in both cytoplasm and nucleus (Figure 5A). To validate the seeded tau aggregation assay, we treated HEK-293FT cells expressing HA-tau_151-391_ with various amounts from 0.375 to 1.5 A.U. of AD O-tau for 42 h and analyzed RIPA-insoluble and -soluble taus by immuno-dot blots developed with anti-HA (Figure 5B). We found that the levels of RIPA-insoluble tau, but not-soluble tau, were increased dose-dependently by AD O-tau (Figure 5C).

We verified RIPA-insoluble tau by Western blots developed with a mixture of R134d & 92e (two polyclonal pan-tau antibodies) and anti-HA. We found that RIPA-insoluble tau in the cells expressing HA-tau_151-391_ and treated with AD O-tau (Figure 5D). Two A.U. of AD O-tau treatment induced more RIPA-insoluble tau than one A.U. of AD O-tau (Figure 5D). Of special note, RIPA insoluble tau displayed multiple bands and SDS- and β-ME-resistant high molecular weight tau (HMW-tau) (Figure 5D, arrowheads). R134d & 92e, but not anti-HA, immuno-reactivity was found in RIPA-insoluble fraction from non-tau-transfected mock cells treated with AD O-tau (Figure 5D), which represented the added AD O-tau. Neither multiple bands nor HMW-tau was observed in RIPA-soluble fraction (Figure 5E), suggesting that seeded tau aggregates were sedimented by ultracentrifugation.

### Assessment of tau seeding activity in AD brain extract and tau_151-391_ aggregates by the seeded tau aggregation assay

**Figure 6:**
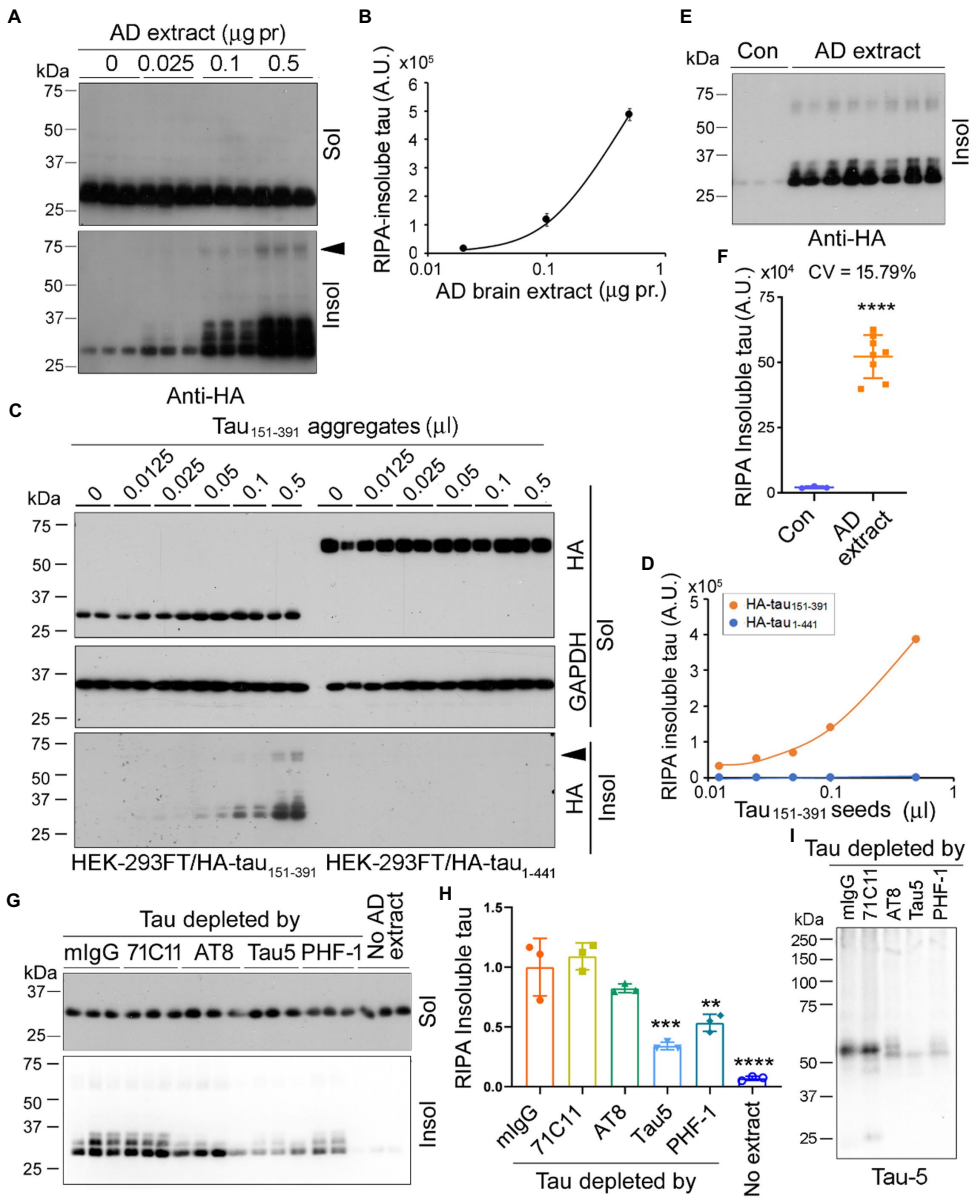
Seed-competent tau from AD brain templates tau_151-391_ aggregation. **(A,B)** AD brain extract seeded tau aggregation. HEK-293FT cells expressing HA-tau_151-391_ in triplicate were treated with various amounts of AD brain extract for 42 h. The RIPA-soluble and -insoluble taus were analyzed **(A)**. RIPA-insoluble tau was plotted against the amount of AD brain extracts **(B)**. **(C,D)** RIPA-insoluble tau aggregates seeded tau aggregation. HEK-293FT cells expressing HA-tau_151-391_ or HA-tau_1-441_ in duplicate were treated with various amounts of tau_151-391_ aggregates for 42 h. The RIPA-soluble and -insoluble taus were analyzed **(C)**. RIPA-insoluble tau was plotted against amounts of tau_151-391_ aggregates **(D)**. **(E,F)** AD brain extract seeded tau aggregation reproducibly. HEK-293FT/HA-tau_151-391_ cells were treated with 0.5 μg protein of AD brain extract in octuplicate. The RIPA-insoluble tau was analyzed, and the coefficient of variation (CV) was calculated. **(G–I)** Depletion of tau from AD brain extract by tau antibody reduced the seeding activity. AD brain extract was incubated with mouse IgG (mIgG) or tau antibodies, 71C11, AT8, Tau5, and PHF-1, pre-coupled onto-protein G beads overnight to deplete tau. HEK-293FT cells expressing HA-tau_151-391_ in triplicate were treated with 0.5 μg protein of brain extract for 42 h. The RIPA-soluble and -insoluble taus were analyzed **(G)**. Relative levels of RIPA-insoluble tau are presented as mean ± SD **(H)**. The brain extracts were subjected to Western blots developed with Tau5 **(I)**. Arrowhead indicates the SDS- and β-ME-resistant HMW-tau species. **p < 0.05; ***p < 0.001; ****p < 0.0001; *vs mIgG; # *vs* no extract.

In addition to AD O-tau, we wanted to know whether tau seeding activity of AD brain extract can be assessed by the seeded tau aggregation assay. Various amounts (0.025, 0.1, 0.5 μg protein) of AD brain extract were used to treat HEK-293FT/HA-tau_151-391_ cells for 42 h. RIPA-insoluble and -insoluble taus were analyzed by Western blots developed with anti-HA (Figure 6A). We found that AD brain extract dose-dependently induced tau_151-391_ aggregation and 0.025 μg protein of AD brain extract was enough to seed tau aggregation (Figure 6B). Similar to AD O-tau, AD brain extract seeded tau aggregates displayed multiple bands and HMW-tau species (Figure 6A).

Tau_151-391_ forms aggregates in cultured HEK-293FT cells (Gu et al., 2020). To learn whether the tau_151-391_ aggregates show seeding activity, we treated HEK-293FT/HA-tau_151-391_ or HEK-293FT/HA-tau_1-441_ cells with various amounts (0.0125–0.5 μL) of tau_151-391_ aggregates for 42 h and detected RIPA-soluble and -insoluble taus (Figure 6C). We found that tau_151-391_ aggregates were able to template HA-tau_151-391_, but not HA-tau_1-441_ aggregation in a dose-dependent manner (Figures 6C,D). These findings are consistent with our previous study where we reported that tau_1-441_ was markedly more resistant to seeded aggregation by AD O-tau than tau_151-391_ (Gu et al., 2020).

We treated HEK-293FT/HA-tau_151-391_ with 0.5 μg protein of AD brain extract in octuplicate for 42 h, RIPA-insoluble tau was determined by Western blots with anti-HA (Figure 6E). Consistently, AD brain extract significantly induced tau_151-391_ aggregation (Figures 6E,F). The coefficient of variation was 15.79%, suggesting good reproducibility.

To learn whether templated tau aggregation is dependent on tau, we incubated AD brain extract with tau antibodies to deplete tau and then treated HEK-293FT/HA-tau_151-391_ cells with the same amounts of the brain extract for 42 h. We found that RIPA-insoluble tau was significantly reduced by the incubation with Tau5 and PHF-1, slightly by AT8 (Figures 6G,H). Western blots confirmed that tau was the most effectively depleted by Tau5 and PHF-1 and partially depleted by AT8 (Figure 6I). These results indicate that in AD brain extract, it was the seeding-competent tau that is responsible for templated tau_151-391_ aggregation.

### Association of tau seeding activity in AD brain assessed by the seeded tau aggregation assay with tau pathology

**Figure 7:**
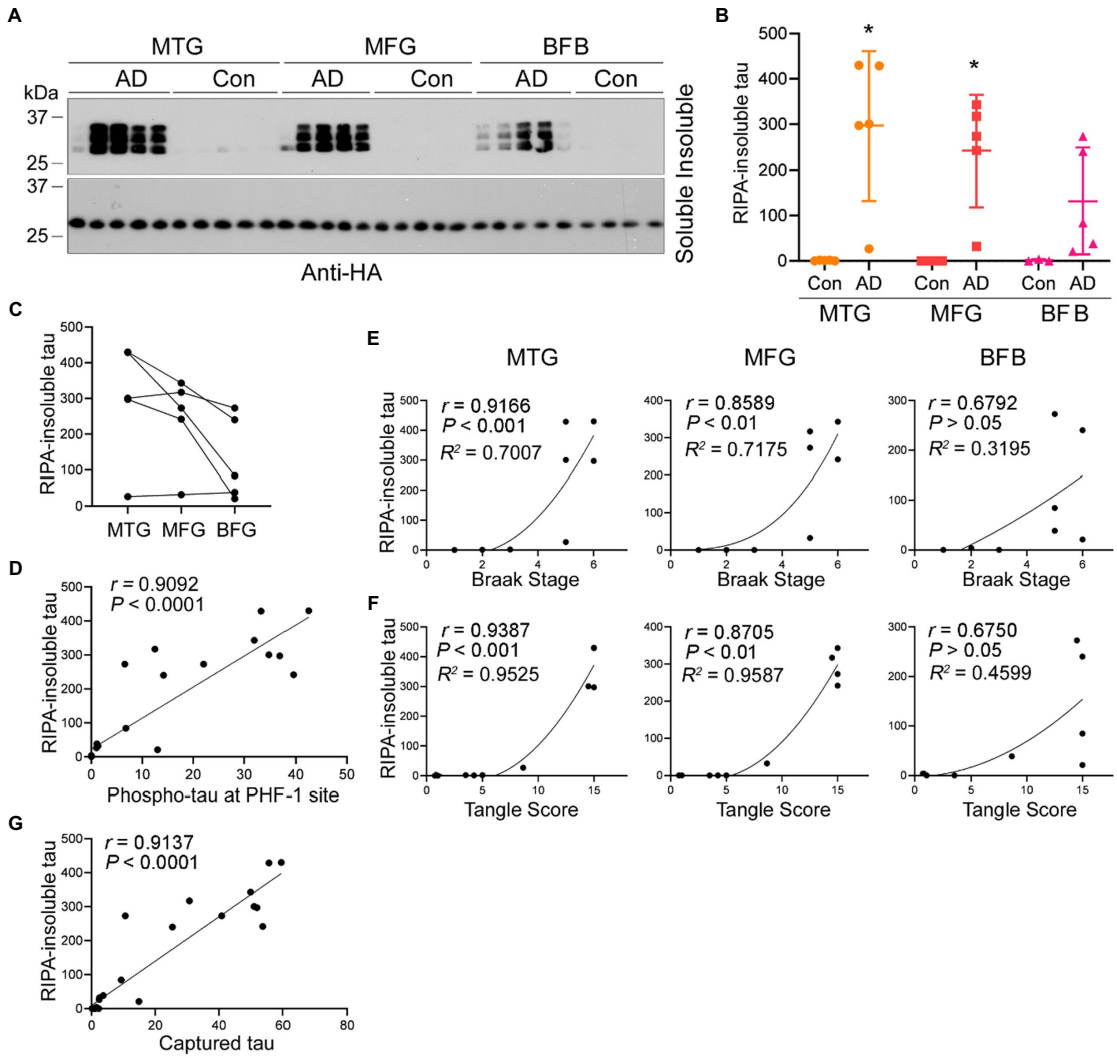
Association of increased seeding activity with tau pathology. **(A–C)** Tau seeding activity in AD brain assessed by the seeded tau aggregation assay. AD brain extracts seeded tau_151-391_ aggregation in cultured cells. HEK-293FT/HA-tau_151-391_ cells were treated with brain extract from three brain regions of AD and control cases for 42 h. RIPA-soluble and -insoluble taus were analyzed by Western blots **(A)** and are presented as scatter plots and mean ± SD **(B)** or across three brain regions of AD cases **(C)**. *, *p* < 0.05. **(D)** Tau seeding activity assessed by the seeded tau aggregation assay was positively correlated with phospho-tau levels in Figure 2C. The levels of RIPA-insoluble tau were plotted against the levels of phospho-tau. The correlation was analyzed by Pearson correlation analysis. **(E,F)** Tau seeding activity assessed by the seeded tau aggregation assay was positively correlated with Braak stage and tangle score. The levels of RIPA-insoluble tau induced by each region were plotted against the Braak stage **(E)** or tangle score **(F)**. The correlation was analyzed by Spearman correlation analysis and *R*^2^ was calculated by non-linear regression. **(G)** Positive correlation of tau seeding activity assessed by two assays. Levels of seeded aggregated tau were plotted against the levels of captured tau. The correlation was analyzed by Pearson correlation analysis.

To assess tau seeding activity in AD brain, the AD brain extracts from MTG, MFG, and BFB as described above were used to treat HEK-293FT/HA-tau_151-391_ cells for 42 h. The RIPA-insoluble and -soluble taus were determined. We found that AD, but not control, brain extracts templated tau_151-391_ aggregation (Figure 7A). Less RIPA-insoluble tau was induced by AD extracts from BFB than the other two regions (Figures 7B,C). The levels of RIPA-insoluble tau were positively correlated with phospho-tau (Figure 7D), Braak stages (Figure 7E) and tangle scores (Figure 7F), suggesting that tau seeding activity is associated with tau pathology in AD brain.

To determine the relationship between tau seeding activity detected by the above two assays, we plotted the levels of RIPA-insoluble aggregated tau against the levels of captured tau by the brain extracts detected above and analyzed the data by Spearman correlation and non-linear regression. The analysis showed that the levels of seeded tau aggregates strongly positively correlated with the levels of captured tau (Figure 7G), suggesting consistent results of both assays in measuring tau seeding activity.

## Discussion

**Figure 8:**
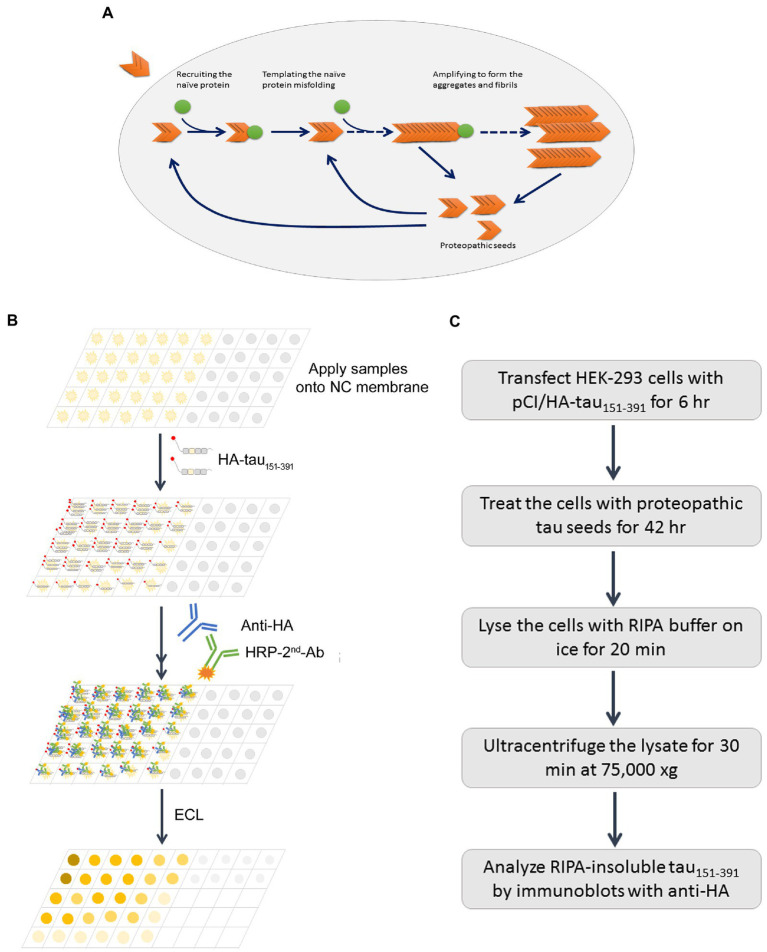
Schematic diagrams of proteopathic tau templated tau aggregation, capture assay, and seeded tau aggregation assay. Misfolded proteopathic tau enters neurons, recruits monomeric/naïve tau and templates its misfolding and aggregation **(A)**. The tau capture assay *in vitro* is based on the capability of preoteopathic tau to recruit naïve tau **(B)**, while the seeded tau aggregation assesses the whole process **(C)**.

Proteopathic tau seeds recruit monomeric tau and template the misfolding and aggregation, underlying the spreading of tau pathology in AD brain (Figure 8). Tau seeding activity in autopsy AD brain is correlated with rate of disease progression and disease severity (Dujardin et al., 2020). The quantification of tau seeding activity in human specimens may be related to the clinical progression of AD and related tauopathies. In this report, we describe two simple assays for assessing tau seeding activity: the *in vitro* tau capture assay (Figure 8B) and in cultured cells the seeded-tau aggregation assay (Figure 8C). Using these two assays, we determined tau seeding activity in MTG, MFG, and BFB of AD and control cases and found that AD brain extracts, but not control brain extracts, captured tau *in vitro* and seeded-tau aggregation in cultured cells expressing tau_151-391_. BFB contained less phospho-tau and displayed less seeding activity. The capability of AD brain extracts to seed tau aggregation was reduced by partial tau depletion with tau antibody. Tau seeding activity was correlated positively with the levels of hyperphosphorylated tau, Braak stages and tangle scores. These two methods are simple, specific, sensitive, and reproducible.

In general, proteopathic seeds recruit, template misfolding and aggregation of naïve tau, leading to the development of seeding activity assays in a wide range. Our capture assay was designed to assess the ability to recruit naïve protein, the first step for the seeded aggregation. Using this assay, we were able to detect tau recruiting activity in down to 315 ng protein of AD brain crude extract, whereas no tau recruiting activity was observed in as much as 5 μg control brain extract. Only brain extracts from AD cases, but not from control cases, captured tau. In addition, heat stable tau from AD brain could not capture tau_151-391_ (Li et al., 2021). Denaturing AD O-tau by boiling in Laemlli sample buffer inhibited its capture ability. These results suggest a high specificity of the assay for measuring the proteopathic tau. Although our assay is not as sensitive as the RT-QulC-based assay that can detect low fg amounts of a given self-propagating tau aggregates (Metrick et al., 2020), the latter assay relies on using heparin to promote the templating of tau monomers and specific technique/instrument. Of note, in addition to assessing tau seeding activity, capture assay can be used to study the impact of post-translational modifications as well as mutations on recruitment by the preopathic tau.

FRET-biosensor assay is the most popular method to evaluate tau seeding activity (Holmes et al., 2014). It relies on HEK-293 cells stably expressing the repeat domain (RD) of tau with P301L mutation fused to GFP (green fluorescence protein) and requires a flow cytometer to perform FRET-flow cytometry (Holmes et al., 2014). FRET-biosensor assay can detect less than ng of the total protein from AD brain extract (Hitt et al., 2021), which is more sensitive than our seeded-tau aggregation assay that requires 25 ng total protein of AD brain extract. AD O-tau or AD brain extract seeded tau aggregation in dose-dependent manner, but no tau_151-391_ aggregation was seeded by brain extracts from control cases. Heat stable tau from AD brain could not seed tau_151-391_ aggregation (Li et al., 2021). The coefficient of variation of seeded tau aggregation assay was 15.79%. Therefore, our seeded-tau aggregation assay is highly specific and reproducible and does not require FRET-flow cytometer.

Different from tau pathology *in vitro*, both cytoplasmic and nuclear tau_151-391_ aggregates seeded by AD O-tau were observed in HeLa cells. Tau is a cytosolic protein, but tau_151-391_ was expressed in both cytoplasm and nucleus. Nucleus aggregation also was be reported in HEK-293 cells expressing tau RD induced by tau fibrils in clone-dependent manner (Sanders et al., 2014; Kaufman et al., 2016), which may help us to understand the molecular mechanism involved in tau aggregation and pathogenesis.

In addition, the capture assay *in vitro* and the seeded-tau aggregation assay in cultured cells can be performed with routine biochemical techniques in a regular biomedical laboratory setting. The levels of captured tau were strongly positively correlated with the levels of RIPA-insoluble tau seeded by the AD brain extracts. Even though these two assays may not ultrasensitive, they can be used to determine the role of mutation and post-translational modifications of tau in the capture and in the seeded-tau aggregates by proteopathic tau (Gu et al., 2020; Wu et al., 2021). Importantly, both assays can offer a platform for drug screening by targeting tau propagation.

In AD brain, tau is hyperphosphorylated and aggregated to form NFTs (Liu et al., 2008; Shi et al., 2008). Dephosphorylation suppresses the seeding activity of AD O-tau *in vitro* (Wu et al., 2021) and *in vivo* (Hu et al., 2016). Here we found that the seeding activity in human brain extract assessed by either the capture assay or the seeded tau aggregation assay was positively correlated with the phospho-tau, Braak stage and tangle score, indicating the association of tau seeding activity with tau pathology. MTG develops tau pathology earlier than MFG (Villemagne et al., 2015). We found here less phospho-tau and seeding activity in MTG than MFG but it did not reach statistical significance. The basal forebrain located in the forebrain to the front of and below the striatum is important in the production of acetylcholine, which is then distributed widely throughout the brain. Cholinergic basal forebrain neurons provide the major cholinergic innervation to the entire cortical mantle, hippocampus, and amygdala (Mesulam et al., 1983; Mesulam and Geula, 1988). Here, we found BFB contained less phospho-tau and less seeding activity compared with MTG and MFG, which might explain the slower development of NFTs in the BFB compared with other cortical regions (Vana et al., 2011).

Amyloid aggregation of protein relies on the formation of β-sheet (Willbold et al., 2021). Here, we found denaturing tau by boiling it in Laemmli buffer, a common method used for SDS-PAGE, reduced the level of captured tau, suggesting that boiling AD O-tau in Laemmli buffer partially killed its seeding activity. Thus, this result supports that β-sheet secondary structure, is required for tau seeding activity, and the denaturants can suppress tau seeding activity.

In summary, we introduce the development of two simple, specific, and reproducible assays for assessing tau seeding activity. Using these two assays, we found high tau seeding activity in the MTG, MFG, and BFB of the AD brain. Tau seeding activity is associated with tau pathology, phospho-tau, Braak stage and tangles score. These two assays are not ultrasensitive, but they can be performed in a regular biomedical laboratory setting with routine biochemical techniques. Of note, they can be used for determining the effect of mutations and post-translational modifications on seeded tau aggregation and for drug screening.

## Data availability statement

The original contributions presented in the study are included in the article/supplementary material, further inquiries can be directed to the corresponding author.

## Ethics statement

Ethical review and approval were not required for the study on human participants in accordance with the local legislation and institutional requirements. Written informed consent for participation was not required for this study in accordance with the national legislation and the institutional requirements.

## Author contributions

FL, RW, NJ, DC, JG, YT, and ZH carried out the study. C-XG and KI helped in discussing the data and editing the manuscript. FL conceived, designed, and supervised the study and wrote the manuscript. All authors contributed to the article and approved the submitted version.

## Funding

This work was supported in part by funds from the New York State Office for People With Developmental Disabilities, and Nantong University and by grants from the U.S. Alzheimer’s Association (DSAD-15-363172), and the National Natural Science Foundation of China (Grants 81872853).

## Conflict of interest

The authors declare that the research was conducted in the absence of any commercial or financial relationships that could be construed as a potential conflict of interest.

## Publisher’s note

All claims expressed in this article are solely those of the authors and do not necessarily represent those of their affiliated organizations, or those of the publisher, the editors and the reviewers. Any product that may be evaluated in this article, or claim that may be made by its manufacturer, is not guaranteed or endorsed by the publisher.
